# Supplementary material for: Exploring the theory, barriers and enablers for patient and public involvement across health, social care and patient safety: a protocol for a systematic review of reviews
Source: BMJ Open. 2017 Oct 24;7(10):e018426. doi: 10.1136/bmjopen-2017-018426 (PMC5665219; doi:10.1136/bmjopen-2017-018426)
Supplement: Supplementary file 2 [file bmjopen-2017-018426supp002.pdf]

Appendix 1: Example search strategy Medline

Searches	Results
consumer participation/	15860
patient participation/	22136
or/1-2	37690
(patient* adj1 (participat* or involv* or engag* or partnership or partners or collaborat* or consult*)).ab,ti.	24412
(public adj1 (participat* or involv* or engag* or partnership or partners or collaborat* or consult*)).ab,ti.	1982
(user* adj1 (participat* or involv* or engag* or partnership or partners or collaborat* or consult*)).ab,ti.	1416
(service user* adj1 (participat* or involv* or engag* or partnership or partners or collaborat* or consult*)).ab,ti.	312
(consumer* adj1 (participat* or involv* or engag* or partnership or partners or collaborat* or consult*)).ab,ti.	895
(lay adj1 (participat* or involv* or engag* or partnership or partners or collaborat* or consult*)).ab,ti.	141
(citizen* adj1 (participat* or involv* or engag* or partnership or partners or collaborat* or consult*)).ab,ti.	404
(carer* adj1 (participat* or involv* or engag* or partnership or partners or collaborat* or consult*)).ab,ti.	281
(caregiver* adj1 (participat* or involv* or engag* or partnership or partners or collaborat* or consult*)).ab,ti.	844
(customer* adj1 (participat* or involv* or engag* or partnership or partners or collaborat* or consult*)).ab,ti.	45
(client* adj1 (participat* or involv* or engag* or partnership or partners or collaborat* or consult*)).ab,ti.	746
(community* adj1 (participat* or involv* or engag* or partnership or partners or collaborat* or consult*)).ab,ti.	7297
(stakeholder* adj1 (participat* or involv* or engag* or partnership or partners or collaborat* or consult*)).ab,ti.	2069
((patient* and public) adj1 (involv* or participat* or engag* or partnership or partners or collaborat* or consult*)).ab,ti.	6090

(user led or user-led or lay control or user control).ab,ti.	201
((representative* or patient representative* or patient advocate* or expert by experience or famil* or relative* or survivor*) adj1 (participat* or involv* or engag* or partnership or partners or collaborat* or consult*)).ab,ti.	6905
((patient* or consumer* or citizen* or advisory) adj1 board*).ab,ti.	1439
((patient* or consumer* or citizen* or advisory) adj1 group*).ab,ti.	75334
((patient* or consumer* or citizen* or advisory) adj1 panel*).ab,ti.	1215
(citizen* adj1 (jury or juries)).ab,ti.	64
or/4-23	128820
3 or 24	159935
health services research.mp. or exp health services research/	151453
social care research.mp.	28
social service*.mp.	8561
public health.mp. or exp public health/	6876537
psychology.mp. or exp psychology/	276350
psychiatry.mp. or exp psychiatry/	122746
sociology.mp. or exp sociology/	1228269
nursing.mp. or exp nursing/	505413
(mental health or mental healthcare).mp. or exp mental health/	135802
secondary care.mp. or exp secondary care/	4285
tertiary care.mp. or exp tertiary healthcare/	33981
or/26-36	8022042
Family Practice/	64218
Primary Health Care/	65710
Physicians, Family/	15936
Community Health Services/	29975
Community Dentistry/	1198
Community Health Nursing/	19280
Community Mental Health Services/	18322
Community Pharmacy Services/	3625
Home Care Services/	31334

Community Mental Health Centers/	2918
family pract$.tw.	8627
general practice$.tw.	34098
community based.tw.	41862
community care.tw.	3486
family medicine.tw.	7419
family physician$.tw.	11393
primary care.tw.	81635
(primary health care or primary healthcare).tw.	18862
family doctor$.tw.	3837
primary medical care.tw.	805
general physician$.tw.	1361
general practitioner$.tw.	40695
primary care practitioner$.tw.	1148
(community adj (health or healthcare or health care)).tw.	16890
primary healthcare team$.tw.	101
primary health care team$.tw.	492
primary medical care team$.tw.	1
practice nurse$.tw.	4672
practice manager$.tw.	374
(gpsi or gpwsi).tw.	30
(practitioner$ adj3 special interest$).tw.	68
(primary care or primary health care or general practice or family practice or family medicine).nw.	40736
or/38-69	369470
health care.mp. or "Delivery of Health Care"/	651145
(health care services or health care delivery).mp.	18161
37 or 71 or 72	8180235
70 or 73	8253391
patient safety.mp. or Patient Safety/	26848
Safety Management/	18778

Medication Errors/ or Patient Harm/ or Medical Errors/	26909
patient risk.mp.	2561
Safety/ or safety.mp.	388412
Adverse event$.mp.	102273
adverse drug event$.mp.	2514
incident$.mp.	96270
error$.mp.	271931
error*.mp.	271931
incident*.mp.	96270
near miss*.mp.	1578
fall*.mp.	196678
slip*.mp.	14834
trip*.mp.	306863
75 or 76 or 77 or 78 or 79 or 80 or 81 or 82 or 83 or 84 or 85 or 86 or 87 or 88 or 89	1288414
74 or 90	8892947
25 and 91	125154
((meta or narrative or bibliometric or systematic) and review).ab,ti.	106442
exp "review"/ or review.ab,ti.	2552853
93 or 94	2552853
92 and 93	2052

Appendix 2: Data Extraction Form

Data Extraction Form	Details
Citation ID (author, year),	
Systematic review aims	
Setting (primary, secondary, social and community care etc)	
Countries where the research conducted	
Health topic focus	
Sample Size and Participant characteristics (younger people, over 65, ethnicity, general public etc) [This should include the final number of studies reported on and participant characteristics (e.g. pre-defined inclusion criteria or characteristics reported on) including sample size if reported]	
Evidence of Equality and Diversity<br><br>*(Equality defined in line with the protected characteristics covered by the Equality &Human Rights Act & NHS Constitution e.g age; disability; gender reassignment; marriage & civil partnership; pregnancy & maternity; race; religion or belief; sex; sexual orientation),*<br><br>**Diversity** literally means difference. When it is used as a contrast or addition to equality, it is about recognising individual as well as group differences, treating people as individuals, and placing positive value on diversity in the community and in the workforce.	
Definition(s) of PPI	
Methods of PPI involvement<br>Involvement can be seen as a spectrum, with a range of service user involvement activities that can take place at multiple levels.<br><br>**Information:** Service users are told what is happening, they have no influence over the decision-making.<br>**Consultation:** Service users are asked their views. They have limited influence decision-making.<br>**Participation:** Service users' views are sought and taken into account. They have a direct impact on decision-making.<br>**Partnership/ co-production**: Working as equals, service users share decisions and responsibility, influencing and determining outcomes.<br>**Control:** Service users control the decision-making.	
Theories, frameworks and concepts and how they are used/defined (e.g. to understand, analyse, describe, facilitate, approach PPI activities)	
Evidence of barriers and facilitators to involvement	
Evidence of impacts of PPI	
