# Supplementary material for: Exploring the theory, barriers and enablers for patient and public involvement across health, social care and patient safety: a protocol for a systematic review of reviews
Source: BMJ Open. 2017 Oct 24;7(10):e018426. doi: 10.1136/bmjopen-2017-018426 (PMC5665219; doi:10.1136/bmjopen-2017-018426)
Supplement: Supplementary file 1 [file bmjopen-2017-018426supp001.pdf]

PRISMA-P (preferred reporting items for systematic review and meta-analysis protocols) checklist: recommended items to address in a systematic review protocol[33]

Section and topic	Item No	Checklist item	Where they are addressed
**Administrative information**			
Title:			
Identification	1a	Identify the report as a protocol of a systematic review	Title page
Update	1b	If the protocol is for an update of a previous systematic review, identify as such	NA
Registration	2	If registered, provide the name of the registry (such as PROSPERO) and registration number	PROSPERO CRD42017067848 (pg.4, study registration)
Authors:			
Contact	3a	Provide name, institutional affiliation, e-mail address of all protocol authors; provide physical mailing address of corresponding author	Title page
Contributions	3b	Describe contributions of protocol authors and identify the guarantor of the review	Page 13
Amendments	4	If the protocol represents an amendment of a previously completed or published protocol, identify as such and list changes; otherwise, state plan for documenting important protocol amendments	NA
Support:			
Sources	5a	Indicate sources of financial or other support for the review	Page 13
Sponsor	5b	Provide name for the review funder and/or sponsor	Health Foundation, NIHR, page 13
Role of sponsor or funder	5c	Describe roles of funder(s), sponsor(s), and/or institution(s), if any, in developing the protocol	NA
**Introduction**			
Rationale	6	Describe the rationale for the review in the context of what is already known	Page 3, second paragraph

Section and topic	Item No	Checklist item	Where they are addressed
Objectives	7	Provide an explicit statement of the question(s) the review will address with reference to participants, interventions, comparators, and outcomes (PICO)	Aim of the review and research question (page 3, second para). This does not include reference to specific participants, interventions comparators and outcomes as the review does not focus on specific interventions.
**Methods**			
Eligibility criteria	8	Specify the study characteristics (such as PICO, study design, setting, time frame) and report characteristics (such as years considered, language, publication status) to be used as criteria for eligibility for the review	Inclusion and Exclusion criteria (page 4)
Information sources	9	Describe all intended information sources (such as electronic databases, contact with study authors, trial registers or other grey literature sources) with planned dates of coverage	Search strategy pages page 5, Table 1
Search strategy	10	Present draft of search strategy to be used for at least one electronic database, including planned limits, such that it could be repeated	Appendix 1: (pages 8-11)
Study records:			
Data management	11a	Describe the mechanism(s) that will be used to manage records and data throughout the review	Screening of studies (page 5 and 6)
Selection process	11b	State the process that will be used for selecting studies (such as two independent reviewers) through each phase of the review (that is, screening, eligibility and inclusion in meta-analysis)	Abstract and Title screening and Full-text screening (page 5)
Data collection process	11c	Describe planned method of extracting data from reports (such as piloting forms, done independently, in duplicate), any processes for obtaining and confirming data from investigators	Data extraction and synthesis (pages 6)

Section and topic	Item No	Checklist item	Where they are addressed
Data items	12	List and define all variables for which data will be sought (such as PICO items, funding sources), any pre-planned data assumptions and simplifications	Data extraction and synthesis (pages 6)
Outcomes and prioritization	13	List and define all outcomes for which data will be sought, including prioritization of main and additional outcomes, with rationale	NA
Risk of bias in individual studies	14	Describe anticipated methods for assessing risk of bias of individual studies, including whether this will be done at the outcome or study level, or both; state how this information will be used in data synthesis	NA
Data synthesis	15a	Describe criteria under which study data will be quantitatively synthesised	NA
	15b	If data are appropriate for quantitative synthesis, describe planned summary measures, methods of handling data and methods of combining data from studies, including any planned exploration of consistency (such as I^2, Kendall's τ)	NA
	15c	Describe any proposed additional analyses (such as sensitivity or subgroup analyses, meta-regression)	NA
	15d	If quantitative synthesis is not appropriate, describe the type of summary planned	Study will be synthesised using a narrative approach (page 6, para 4).
Meta-bias(es)	16	Specify any planned assessment of meta-bias(es) (such as publication bias across studies, selective reporting within studies)	NA
Confidence in cumulative evidence	17	Describe how the strength of the body of evidence will be assessed (such as GRADE)	NA
