# Supplementary material for: Exploring the theory, barriers and enablers for patient and public involvement across health, social care and patient safety: a protocol for a systematic review of reviews
Source: BMJ Open. 2017 Oct 24;7(10):e018426. doi: 10.1136/bmjopen-2017-018426 (PMC5665219; doi:10.1136/bmjopen-2017-018426)
Supplement: Author's manuscript [file bmjopen-2017-018426.draft_revisions.pdf]

BMJ Open

BMJ Open is committed to open peer review. As part of this commitment we make the peer review history of every article we publish publicly available.

When an article is published we post the peer reviewers' comments and the authors' responses online. We also post the versions of the paper that were used during peer review. These are the versions that the peer review comments apply to.

The versions of the paper that follow are the versions that were submitted during the peer review process. They are not the versions of record or the final published versions. They should not be cited or distributed as the published version of this manuscript.

BMJ Open is an open access journal and the full, final, typeset and author-corrected version of record of the manuscript is available on our site with no access controls, subscription charges or pay-per-view fees (http://bmjopen.bmj.com).

If you have any questions on BMJ Open's open peer review process please email editorial.bmjopen@bmj.com

BMJ Open

Exploring the theory, barriers and enablers for patient and public involvement across health, social care and patient safety: A protocol for a systematic review of reviews.

Journal:	*BMJ Open*
Manuscript ID	bmjopen-2017-018426
Article Type:	Protocol
Date Submitted by the Author:	30-Jun-2017
Complete List of Authors:	Ocloo, J; King's College London, Health Service & Population Research Department<br>Garfield, Sarah; Imperial College Healthcare NHS Trust, Centre for Medication Safety and Service Quality; University College London, School of Pharmacy<br>Dawson, Shoba; University College London Ear Institute, evidENT<br>Dean Franklin, Bryony; University College London School of Pharmacy; Imperial College Healthcare NHS Trust, Centre for Medication Safety and Service Quality
**Primary Subject Heading**:	Health services research
Secondary Subject Heading:	Evidence based practice
Keywords:	patient and public involvement, patient safety and quality, health and social care, theory, barriers and enablers

SCHOLARONE™ Manuscripts

For peer review only

1

Title Page

Exploring the theory, barriers and enablers for patient and public involvement across health, social care and patient safety: A protocol for a systematic review of reviews.

Dr Josephine Ocloo, Dr Sara Garfield, Ms Shoba Dawson, Prof Bryony Dean Franklin

Corresponding author:

Dr Josephine Ocloo, Centre for Implementation Science (CIS) part of the National Institute for Health Research (NIHR) Collaboration for Leadership in Applied Health Research and Care South London.
Health Service & Population Research Department
Institute of Psychiatry, Psychology & Neuroscience (IoPPN)
King's College London Room M306De Crespigny Park
Denmark Hill
London SE5 8AF
UK.
Email: Josephine.ocloo@kcl.ac.uk
Tel: +44 0 207 848 0115

Co—authors:

Dr Sara Garfield, Imperial College Healthcare NHS Trust, London, UK,
University College London School of Pharmacy, London, UK.

Ms Shoba Dawson
University College London Ear Institute, evidENT
London, UK.

Professor Bryony Dean Franklin, Imperial College Healthcare NHS Trust, London, UK
University College London School of Pharmacy
London, UK .

Key words:

Patient and public Involvement, theory barriers and enablers, patient safety and quality, health and social care.

Word count: 2,467 (excluding title page appendices and references)

Peer review only

Exploring the theory, barriers and enablers for patient and public involvement across health, social care and patient safety: A protocol for a systematic review of reviews.

Abstract

Introduction: The emergence of patient and public involvement (PPI) in healthcare in the UK can be traced as far back as the 1970's. More recently, campaigns by harmed patients and their relatives have emerged as a result of clinical failings in the NHS, challenging paternalistic healthcare, which have led to a new focus on PPI in quality and safety, nationally and internationally. Evidence suggests that PPI within patient safety is often atheoretical and located within a biomedical discourse. This review will explore the literature on PPI across patient safety, healthcare and social care to identify theory, barriers and enablers from other parts of the public sector that can be used to develop PPI in patient safety.

Methods and analysis: Systematic searches of three electronic bibliographic databases will be conducted, using both MeSH and free-text terms to identify empirical literature published from database inception to 2017. The screening process will involve input from at least two researchers and any disagreement will be resolved through discussion with a third reviewer. Initial inclusion and exclusion criteria have been developed and will be refined iteratively throughout the process. Data extraction from included articles will be conducted by at least two researchers using a data extraction form. Extracted information will be analysed using a narrative review approach, which synthesises data using a descriptive method.

Conclusion: This review is important given the current renewed focus on PPI in quality and safety improvement and research in healthcare. Identifying theory, barriers and enablers to PPI from other parts of the public sector will potentially be important in empowering patients and the public in patient safety. The findings of this review will provide important opportunities for testing methods of PPI in patient safety and evaluating their impact in improving services.

Keywords: patient and public Involvement, theory, barriers and enablers, patient safety and quality, health and social care.

For peer review only

Strengths and limitations of this study

This systematic review protocol makes an original contribution by identifying and synthesising evidence from across health and social care and patient safety to provide an overview of the theory, barriers and enablers for undertaking patient and public involvement (PPI). The evidence identified will be used to develop approaches to PPI in patient safety, which is more underdeveloped, than in other parts of healthcare and the wider public sector. Undertaking a review of reviews also allows us to highlight and bring together information identified about the topic under study, which can be used to provide recommendations to inform clinical practice.

The protocol complies with a well-established systematic review methodology that is transparent and replicable and based on internationally recognised standards. A limitation of this review is that it does not cover the grey literature. The review will therefore focus solely on published empirical peer-reviewed reviews in health and social care and the patient safety context, but we believe the findings and recommendations will be relevant for their intended audience of academics and policy makers.

Introduction:

The importance of involving patients, service users, carers and the public in the UK in health and social care and research has grown significantly in recent decades[1-4]. These developments have been linked to the growth in policy initiatives around citizenship, democracy and rights[5]. More recently the idea of partnership working with patients and the public has gained even more prominence as a result of serious clinical and service failings in the UK[6,7], and internationally[8-10]. These initiatives have often been driven by the campaigns of patients who have experienced harm and their relatives, leading to a new focus on PPI and its importance in improving quality and safety within healthcare.

In the United Kingdom, a commitment to patient and public involvement (PPI) is now firmly enshrined in key legislation. This covers the Health and Social Care Act[11], the NHS Constitution[12] and the duty by NHS England (s13Q of the National Health Service Act 2006 (as amended by the Health and Social Care Act 2012) to properly involve patients and the public in its commissioning processes and decisions. In addition, key regulations set out essential standards of quality and safety that people who use health and adult social care services have a right to expect[13]. These rights are underpinned by wider but complimentary policy approaches such as public and patient experience and engagement (PPE). These approaches aim to place people who use services at the centre of care, to understand their experience of services, to empower them to make decisions and involve them in the design and delivery of care[14]. The NHS next stage review[15] identifies three strands of quality: patient experience, patient safety and clinical effectiveness. PPE approaches aim to ensure that patient experience sits as an equal partner in these three strands of quality[15].

These types of PPI initiatives are by no means unique to the UK and similar reforms in health and social care can be seen across a range of international settings[16-20].

While there is no general consensus on defining PPI, it has increasingly come to be seen as a process of partnership: *"…the active participation of patients, carers, community representatives, community groups and the public in how services are planned, delivered and evaluated. It is broader and deeper than traditional consultation. It involves the ongoing process of developing and sustaining constructive relationships, building strong, active partnerships and holding a meaningful dialogue*

For peer review only

with stakeholders. Effective engagement leads to improvements in health services and is part of everyone's role in the NHS." (p.9)[13]

Despite this understanding, there have been an increasing number of criticisms about the nature of PPI. There is uncertainty about how to do it well, in ways that constitute genuine partnerships and which involve a diversity of patients and the public, rather than a few selected individuals[21-23]. In particular, in the area of patient safety, the literature on PPI has been seen as dominated by a biomedical approach[24,25], has been atheoretical[26] and has not addressed power inequities and discrimination[27]. This has exposed PPI to criticisms of exclusivity and tokenism[28].

Given this context, this review is being undertaken to address a perceived knowledge and theory gap with PPI in patient safety. This gap is seen as leading to a narrow model of PPI that fails to empower patients and the public in the involvement process. This gap is seen to be out of keeping with the wider literature on PPI more broadly in healthcare, which highlights the contested and bottom up nature and drivers for involvement and the way in which various global health social movements have provided collective challenges to poor care and discriminatory or paternalistic services and medical policy and belief systems[29]. These drivers have led to the development of theory, methods and approaches, particularly within mental health, that have been used to develop wider PPI models and methods based upon co-production and partnership. In patient safety, despite the campaigns by patients who have experienced harm and their relatives acting as a catalyst for the patient safety movement, there is evidence that suggests that patient representatives are struggling to influence decisions and are largely expected to work within existing systems in improving quality and safety[7,26]. Involvement at this level has therefore been criticised as providing little opportunity to influence decision-making processes in any depth, maintaining power differentials and the status quo.

Therefore the aim of this review is to identify reviews of the published empirical literature across health and social care and patient safety to understand the theory, barriers and enablers in undertaking PPI, which can be used to inform PPI in patient safety.

Methods and analyses

Study design

This systematic review of reviews will be conducted and reported in accordance with the Preferred Reporting Items for Systematic review and Meta-Analysis Protocols (PRISMA-P) (see Research Checklist)[32] and the results reported following the PRISMA guidelines[33]. The flow diagram (Figure 1) demonstrates the different stages of the systematic review and how these interact and influence one another.

Flow Diagram – Stages of the Systematic Review

Insert Figure 1

Study registration

Based on the PRISMA-P guidelines, this review was registered with the International Prospective Register of Systematic Reviews (PROSPERO) on 28/4/2017 (registration number CRD42017067848: (http://www.crd.york.ac.uk/PROSPERO/).

Eligibility criteria

For peer review only - http://bmjopen.bmj.com/site/about/guidelines.xhtml

Studies will be included in this review if they fulfil the following criteria:

Inclusion Criteria

1. **Type of study** – systematic reviews that focus on the concept of, or approaches to, PPI and/or PPE across patient safety, healthcare and social care.
2. **Setting** - any organisational setting (e.g. primary care, mental health, hospital, tertiary care, and voluntary etc)
3. **Type of involvement -** This review will focus largely on the collective level or what has also been referred to as public involvement [e.g. in service improvement or planning and organisational design, governance, policy making, commissioning, monitoring, evaluation and research]. The review will however also look at some examples of involvement in direct care where this relates to activities to improve health, social care or patient safety and quality more widely. The review will not look at the much wider and more substantial body of literature on aspects or proxies for patient involvement, or engagement in their own treatment such as shared decision- making and patient centeredness.
4. **Study design** - systematic reviews based on either published empirical studies (e.g. using quantitative, qualitative, or mixed methods), or theoretical papers. Where papers include both a systematic review and an empirical study, we will include data relating to the review if it is presented separately.

Articles that meet any of the following criteria will be excluded:

1. Systematic reviews that do not have a specific focus on PPI at the collective service improvement level.
2. Empirical studies
3. Non-systematic reviews
4. Reviews that focus on PPI at the individual level in treatment and decision-making.
5. Reviews not written in English

Search strategy

Three main electronic bibliographic databases including MEDLINE, EMBASE and PsycINFO will be searched for potential studies from the inception of databases to May 2017 as described in Table 1.

Table 1: A structured search of key electronic bibliographic databases:

Search type:	Database:	Database history:	Filters applied:
Systematic review of the literature	(MEDLINE (via OVID 1946 to May 2017)	Biomedical database produced by the National Library of Medicine, with over 3000 journals	Limited to systematic reviews, narrative reviews, bibliometric reviews and meta-analysis
	EMBASE (1974 to May 2017	Biomedical and pharmacological database, with a more European focus	Limited to systematic reviews, narrative reviews, bibliometric reviews and meta

For peer review only

6

	PsycINFO (1967 to May 2017).	Produced by the American Psychological Association covering over 1300 journals from Psychology and related disciplines	Limited to systematic reviews, narrative reviews, bibliometric reviews and meta

A comprehensive search strategy will include a combination of five main blocks of terms including and relating to public involvement **(**public, patient, carer, consumer, citizen, lay, service user, stakeholder, family, relative, survivor); type of involvement (involvement, collaboration, engagement, partnership, consultation, participation, user-led, consumer or patient panel, advisory board/group/panel); health and social care setting (health services, health care, social care, public health, mental health etc); patient safety (safe*, adverse event$, error* etc) and type of review (systematic, narrative, meta, bibliometric); using a combination of MeSH (Medical Subject Headings) terms and free text (Appendix 1: example search strategy). This will be supplemented by citation searches and reference lists of included studies. Additionally, several scoping exercises in these electronic databases will be applied to maximise the sensitivity and specificity of the developed search strategy.

Screening of studies

An Endnote library will be utilised to combine and export the results of the searches from different databases. Duplicates will be removed prior to the selection of studies. Study selection will be completed in two stages. Firstly, titles and abstracts will be screened in order to identify eligible and relevant studies, followed by full-text screening to identify reviews eligible for inclusion.

Abstract and title screening

Abstracts and titles will be used to initially screen and identify eligible and relevant studies using the selection criteria above for the articles remaining in the literature review. A random sample of 10% titles and abstracts will be independently screened by both the lead researcher and a secondary reviewer (SG or SD). Any disagreements will be resolved through discussions until consensus is reached. However, if we are unable to reach consensus, disagreements will be resolved through discussions with an independent third reviewer (BDF). Once a consensus is reached for the 10% sample, the remaining titles and abstracts will be screened by the lead researcher alone.

Full-text screening

At the outset of this process a 10% sample of full-text papers will be screened and reviewed independently by both the lead researcher and a second reviewer (SG or SD). Any disagreements will be resolved through discussion or involving a third reviewer. If necessary a further 10% of papers will be independently reviewed to test for agreement and then the remaining reviews will be screened by the lead researcher (JO) alone. Along with the lead researcher, SG and SD will screen all included papers to ensure they meet the inclusion criteria.

Data extraction and synthesis:

A data extraction form will be used (Appendix 2), to focus on the characteristics that are relevant to this study. The form will be piloted on 10% of randomly selected studies. Any necessary modifications will then be made to the data extraction tool. Data from the included studies will be extracted and categorised using the following headings below.

For peer review only

7

- Study characteristics- authors, year, study aims, type of study/study design, health topic focus and setting
- Participant characteristics- types of people involved (including age, ethnicity, gender etc)
- Evidence of equality and diversity (in accordance with the Equality and Human Rights Act and NHS Constitution)
- Definition(s) of PPI
- Methods of PPI (i.e. consultation, collaboration or user control)
- Use of theory (to understand, analyse, describe, and/or facilitate PPI activities)
- Barriers and facilitators to involvement.
- Reported impacts of PPI

The data extraction process will be carried out by both the lead researcher and a secondary reviewer (SG or SD) as per the earlier processes. Any disagreements will be resolved through discussion or involving a third reviewer.

A systematic narrative approach will be used for synthesising the results of this review, due to the heterogeneous nature of the included published empirical literature.[34] This will include familiarisation with the papers and identifying data relating to the themes linked to study aims and outcomes. Data will be summarised in a descriptive form in order to allow us to draw conclusions about the available evidence.

Quality assessment

A quality assessment is normally conducted in systematic reviews using quality assessment checklists such as Critical Appraisal Skills Programme[1] (CASP) to assess the quality of individual studies. As this is a systematic review of reviews, an empirically developed instrument for documenting the methodological quality of systematic reviews: Assessment of Methodological Quality of Systematic Reviews (AMSTAR)[2], will be used in this study to determine if eligible reviews meet the minimum criteria based on quality.

Conclusion

To our knowledge this is the first systematic review of reviews investigating PPI in health and social care and patient safety. Previous reviews have tended to focus on one sector specifically, rather than look at PPI across all three sectors, with a view to informing the development of good practice. This review of reviews is important given the current renewed policy focus on PPI in quality and safety improvement and research in healthcare. Identifying the theory, barriers and enablers from parts of the public sector that have been more successful in developing involvement processes will be important in helping to develop PPI practice that is less well developed in areas such as patient safety. The findings of this review will be used in the future to construct a theory driven framework with respect to PPI that can be used to support the testing of different interventions of PPI in patient safety and evaluate its impact.

---

[1] http://www.casp-uk.net/casp-tools-checklists

[2] https://amstar.ca/Amstar_Checklist.php

For peer review only

8

Appendix 1: Example search strategy Medline

Searches	Results
consumer participation/	15860
patient participation/	22136
or/1-2	37690
(patient* adj1 (participat* or involv* or engag* or partnership or partners or collaborat* or consult*)).ab,ti.	24412
(public adj1 (participat* or involv* or engag* or partnership or partners or collaborat* or consult*)).ab,ti.	1982
(user* adj1 (participat* or involv* or engag* or partnership or partners or collaborat* or consult*)).ab,ti.	1416
(service user* adj1 (participat* or involv* or engag* or partnership or partners or collaborat* or consult*)).ab,ti.	312
(consumer* adj1 (participat* or involv* or engag* or partnership or partners or collaborat* or consult*)).ab,ti.	895
(lay adj1 (participat* or involv* or engag* or partnership or partners or collaborat* or consult*)).ab,ti.	141
(citizen* adj1 (participat* or involv* or engag* or partnership or partners or collaborat* or consult*)).ab,ti.	404
(carer* adj1 (participat* or involv* or engag* or partnership or partners or collaborat* or consult*)).ab,ti.	281
(caregiver* adj1 (participat* or involv* or engag* or partnership or partners or collaborat* or consult*)).ab,ti.	844
(customer* adj1 (participat* or involv* or engag* or partnership or partners or collaborat* or consult*)).ab,ti.	45
(client* adj1 (participat* or involv* or engag* or partnership or partners or collaborat* or consult*)).ab,ti.	746
(community* adj1 (participat* or involv* or engag* or partnership or partners or collaborat* or consult*)).ab,ti.	7297
(stakeholder* adj1 (participat* or involv* or engag* or partnership or partners or collaborat* or consult*)).ab,ti.	2069
((patient* and public) adj1 (involv* or participat* or engag* or partnership or partners or collaborat* or consult*)).ab,ti.	6090

9

(user led or user-led or lay control or user control).ab,ti.	201
((representative* or patient representative* or patient advocate* or expert by experience or famil* or relative* or survivor*) adj1 (participat* or involv* or engag* or partnership or partners or collaborat* or consult*)).ab,ti.	6905
((patient* or consumer* or citizen* or advisory) adj1 board*).ab,ti.	1439
((patient* or consumer* or citizen* or advisory) adj1 group*).ab,ti.	75334
((patient* or consumer* or citizen* or advisory) adj1 panel*).ab,ti.	1215
(citizen* adj1 (jury or juries)).ab,ti.	64
or/4-23	128820
3 or 24	159935
health services research.mp. or exp health services research/	151453
social care research.mp.	28
social service*.mp.	8561
public health.mp. or exp public health/	6876537
psychology.mp. or exp psychology/	276350
psychiatry.mp. or exp psychiatry/	122746
sociology.mp. or exp sociology/	1228269
nursing.mp. or exp nursing/	505413
(mental health or mental healthcare).mp. or exp mental health/	135802
secondary care.mp. or exp secondary care/	4285
tertiary care.mp. or exp tertiary healthcare/	33981

10

or/26-36	8022042
Family Practice/	64218
Primary Health Care/	65710
Physicians, Family/	15936
Community Health Services/	29975
Community Dentistry/	1198
Community Health Nursing/	19280
Community Mental Health Services/	18322
Community Pharmacy Services/	3625
Home Care Services/	31334
Community Mental Health Centers/	2918
family pract$.tw.	8627
general practice$.tw.	34098
community based.tw.	41862
community care.tw.	3486
family medicine.tw.	7419
family physician$.tw.	11393
primary care.tw.	81635
(primary health care or primary healthcare).tw.	18862
family doctor$.tw.	3837

For peer review only - http://bmjopen.bmj.com/site/about/guidelines.xhtml

11

primary medical care.tw.	805
general physician$.tw.	1361
general practitioner$.tw.	40695
primary care practitioner$.tw.	1148
(community adj (health or healthcare or health care)).tw.	16890
primary healthcare team$.tw.	101
primary health care team$.tw.	492
primary medical care team$.tw.	1
practice nurse$.tw.	4672
practice manager$.tw.	374
(gpsi or gpwsi).tw.	30
(practitioner$ adj3 special interest$).tw.	68
(primary care or primary health care or general practice or family practice or family medicine).nw.	40736
or/38-69	369470
health care.mp. or "Delivery of Health Care"/	651145
(health care services or health care delivery).mp.	18161
37 or 71 or 72	8180235
70 or 73	8253391
patient safety.mp. or Patient Safety/	26848
Safety Management/	18778

12

Medication Errors/ or Patient Harm/ or Medical Errors/	26909
patient risk.mp.	2561
Safety/ or safety.mp.	388412
Adverse event$.mp.	102273
adverse drug event$.mp.	2514
incident$.mp.	96270
error$.mp.	271931
error*.mp.	271931
incident*.mp.	96270
near miss*.mp.	1578
fall*.mp.	196678
slip*.mp.	14834
trip*.mp.	306863
75 or 76 or 77 or 78 or 79 or 80 or 81 or 82 or 83 or 84 or 85 or 86 or 87 or 88 or 89	1288414
74 or 90	8892947
25 and 91	125154
((meta or narrative or bibliometric or systematic) and review).ab,ti.	106442
exp "review"/ or review.ab,ti.	2552853
93 or 94	2552853
92 and 93	2052

For peer review only - http://bmjopen.bmj.com/site/about/guidelines.xhtml

13

Appendix 2: Data Extraction Form

Data Extraction Form	Details
Citation ID (author, year),	
Systematic review aims	
Setting (primary, secondary, social and community care etc)	
Countries where the research conducted	
Health topic focus	
Sample Size and Participant characteristics (younger people, over 65, ethnicity, general public etc) [This should include the final number of studies reported on and participant characteristics (e.g. pre-defined inclusion criteria or characteristics reported on) including sample size if reported]	
Evidence of Equality and Diversity<br><br>*(Equality defined in line with the protected characteristics covered by the Equality &Human Rights Act & NHS Constitution e.g age; disability; gender reassignment; marriage & civil partnership; pregnancy & maternity; race; religion or belief; sex; sexual orientation),*<br><br>**Diversity** literally means difference. When it is used as a contrast or addition to equality, it is about recognising individual as well as group differences, treating people as individuals, and placing positive value on diversity in the community and in the workforce.	
Definition(s) of PPI	
Methods of PPI involvement<br>Involvement can be seen as a spectrum, with a range of service user involvement activities across it. A spectrum of involvement adapted from Arnstein's Ladder is shown below.<br>**Information:** Service users are told what is happening, they have no influence over the decision-making.<br>**Consultation:** Service users are asked their views. They have limited influence decision-making.<br>**Participation:** Service users' views are sought and taken into account. They have a direct impact on decision-making.<br>**Partnership/ co-production**: Working as equals, service users share decisions and responsibility, influencing and determining outcomes.<br>**Control:** Service users control the decision-making.	
Theories, frameworks and concepts and how they are used/defined (e.g. to understand, analyse, describe, facilitate, approach PPI activities)	
Evidence of barriers and facilitators to involvement	
Evidence of impacts of PPI	

For peer review

14

Contributorship Statement

The first author developed the idea for the review and led on all of the work. The second author contributed to the development of the protocol and paper for publication. The third author contributed to the search strategy, to the protocol and paper for publication and the last author acted as an independent person in helping to resolve disagreement and reach consensus and contributed to the protocol and paper for publication.

Competing Interests

None

Funding

The Health Foundation, National Institute for Health Research (NIHR)

Disclaimer

The research is supported by the Centre for Implementation Science (CIS) which is part of the National Institute for Health Research (NIHR) Collaboration for Leadership in Applied Health Research and Care South London at King's College Hospital NHS Foundation Trust and the National Institute for Health Research (NIHR) Imperial Patient Safety Translational Research Centre. The views expressed are those of the author(s) and not necessarily those of the NHS, the NIHR or the Department of Health.

References

1. Department of Health. Patient and Public Involvement in the NHS. London: Department of Health. 1999.

2. Department of Health. Real Involvement: Working with People to Improve Services. London: Department of Health. 2008.

3. Department of Health. Equity and Excellence: Liberating the NHS. London: Department of Health. 2010.

4. Department of Health. Framework for patient and public participation in public health commissioning. London: Department of Health. 2017.

5. Newman J. Modernising Governance: New Labour, Policy and Society. London: Sage; 2001.

6. Bristol Royal Infirmary Inquiry. The Report of the Public Inquiry into Children's Heart surgery at the Bristol Royal Infirmary 1984–1995. London: HMSO, 2001.

7. Francis R. Report of the Mid Staffordshire NHS Foundation Trust Public Inquiry. London: Stationery Office, 2013.

8. Kohn LT, Corrigan JM, Donaldson MS. To err is human: building a safer health system. Washington: National Academies Press, 1999.

9. Walshe K, Shortell SM. When things go wrong: how Health Care Organizations deal with major failures. Health Aff (Millwood) 2004; 23: 103–11.

For peer review only

10. WHO. World Alliance for Patient Safety forward programme. Geneva: World Health Organization, 2008–2009.

11. Department of Health. Research Governance Framework for Health and Social Care. 2005. https://www.gov.uk/government/publications/research-governance-framework-for-health-and-social-care-second-edition. Accessed 12 Jan 2017.

12. Department of Health. NHS Constitution for England. London: Department of Health. 2012. https://www.gov.uk/government/publications/the-nhs-constitution-for-england

13. Care Quality Commission: Guidance about compliance Essential standards of quality and safety March 2010.

14. Department of Health. Putting people at the heart of care. London: Department of Health. 2009. http://webarchive.nationalarchives.gov.uk/20130107105354/http:/www.dh.gov.uk/en/PublicationsandStatistics/Publications/PublicationsPolicyAndGuidance/DH_106038

15. Darzi A. High Quality Care For All: NHS Next Stage Review Final Report. London: Department of Health. 2009.

16. Charles C and DeMaio S. Lay participation in health care decision-making: a conceptual framework. Journal of Health Politics, Policy and Law, 1993

17. Church J, Saunders D, Wanke M et al. Citizen participation in health decision-making: past experience and future prospects. Health Policy 2002; 23:12–32.

18. Tritter J.Q. Revolution or evolution: the challenges of conceptualizing patient and public involvement in a consumerist world, Health Expectations, 2009; 12:3, 275–87.

19. Coulter, A. Engaging Patients in Healthcare. New York: McGraw Hill Professional. 2013.

20. Carman KL, Dardess P, Maurer M et al. Patient and family engagement: a framework for understanding the elements and developing interventions and policies. Health Affairs (Millwood) 2013; 32:223–31.

21. National Institute for Health Research. Going the Extra Mile: improving the Nation's health and wellbeing through public involvement in research. London: National Institute for Health Research, 2015.

22. Beresford P. Beyond the usual suspects. London: Shaping Our Lives. 2013.

23. Martin GP. Representativeness, legitimacy and power in public involvement in health-service management. Social Science and Medicine 2008; 67:1757–65.

24. Sharpe VA and Faden AI. Medical Harm. Cambridge: Cambridge University Press. 2001.

25. Ocloo JE. Harmed patients gaining voice: challenging dominant perspectives in the construction of medical harm and patient safety reforms. Social Science and Medicine 2010; 71:510–16.

For peer review only

26. Peat M, Entwistle V, Hall J, Birks Y, Golder G. Scoping review and approach to appraisal of interventions intended to involve patients in patient safety. Journal of Health Services Research & Policy, 2010; 15: 17–25.

27. Ocloo JE, Fulop NJ. Developing a critical approach to patient and public involvement in patient safety in the NHS: learning lessons from other parts of the public sector? Health Expect 2012; 15:424–32.

28. Ocloo J, Matthews R. BMJ Quality and Safety 2016; 0:1–7. doi:10.1136/bmjqs-2015-004839

29. Brown P, Zavestoski S. Social movements in health: an introduction. Sociology of Health and Illness 2004; 26: 679–94.

30. Patient and Public Engagement Toolkit for World Class Commissioning (NHS South Central WCC Collaborative PPI Programme): www.engage.hscni.net/library/PPE%20Toolit%20-%20South%20Central.pdf

31. Faulkner A, Yiannoullou S, Kalathil J. et al. Involvement for Influence. 4PI National Involvement Standards. London: National survivor User Network (NSUN), 2015.

32. Shamseer L, Moher D, Clarke M. et al. PRISMA-P Group. Preferred reporting items for systematic review and meta-analyses protocols (PRISMA-P) 2015: elaboration and explanation. BMJ. 2015; 349 :g7647. doi:10.1136/bmj.g7647

33. Moher D, Liberati A, Tetzlaff J, Altman DG, The PG. Preferred Reporting Items for Systematic Reviews and Meta-Analyses: The PRISMA Statement. PLoS Medicine. 2009; 6(7):e1000097.

34. Popay J, Roberts H, Sowden A, Petticrew M, Arai L, Rodgers M, Britten N, Roen K and Duffy S. Guidance on the Conduct of Narrative Synthesis in Systematic Reviews: A Product from the ESRC Methods Programme. (2006).

For peer review only

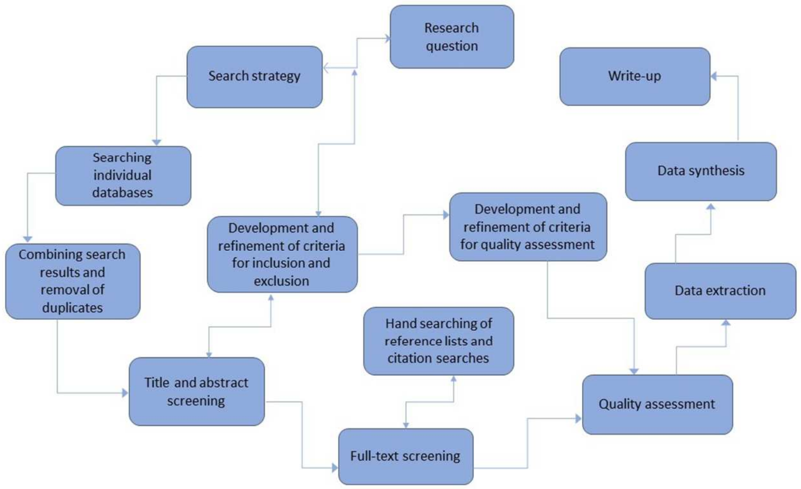

Flow Diagram – Stages of the Systematic Review

338x190mm (96 x 96 DPI)

review only

PRISMA-P (preferred reporting items for systematic review and meta-analysis protocols) checklist: recommended items to address in a systematic review protocol[32]

Section and topic	Item No	Checklist item	Where they are addressed
**Administrative information**			
Title:			
Identification	1a	Identify the report as a protocol of a systematic review	Title page (Page 1)
Update	1b	If the protocol is for an update of a previous systematic review, identify as such	NA
Registration	2	If registered, provide the name of the registry (such as PROSPERO) and registration number	PROSPERO CRD42017067848 (pg.4, study registration)
Authors:			
Contact	3a	Provide name, institutional affiliation, e-mail address of all protocol authors; provide physical mailing address of corresponding author	Title page (Page 1)
Contributions	3b	Describe contributions of protocol authors and identify the guarantor of the review	Page 14
Amendments	4	If the protocol represents an amendment of a previously completed or published protocol, identify as such and list changes; otherwise, state plan for documenting important protocol amendments	NA
Support:			
Sources	5a	Indicate sources of financial or other support for the review	Page 14
Sponsor	5b	Provide name for the review funder and/or sponsor	Health Foundation, NIHR, page 14
Role of sponsor or funder	5c	Describe roles of funder(s), sponsor(s), and/or institution(s), if any, in developing the protocol	NA

For peer review only - http://bmjopen.bmj.com/site/about/guidelines.xhtml

Section and topic	Item No	Checklist item	Where they are addressed
**Introduction**			
Rationale	6	Describe the rationale for the review in the context of what is already known	Page 4, third paragraph
Objectives	7	Provide an explicit statement of the question(s) the review will address with reference to participants, interventions, comparators, and outcomes (PICO)	Aim of the review (page 4)
**Methods**			
Eligibility criteria	8	Specify the study characteristics (such as PICO, study design, setting, time frame) and report characteristics (such as years considered, language, publication status) to be used as criteria for eligibility for the review	Inclusion and Exclusion criteria (page 5)
Information sources	9	Describe all intended information sources (such as electronic databases, contact with study authors, trial registers or other grey literature sources) with planned dates of coverage	Search strategy pages 5-6 (Table 1)
Search strategy	10	Present draft of search strategy to be used for at least one electronic database, including planned limits, such that it could be repeated	Appendix 1: (pages8-12)
Study records:			
Data management	11a	Describe the mechanism(s) that will be used to manage records and data throughout the review	Screening of studies (page 6)
Selection process	11b	State the process that will be used for selecting studies (such as two independent reviewers) through each phase of the review (that is, screening, eligibility and inclusion in meta-analysis)	Abstract and Title screening and Full-text screening (page 6)
Data collection process	11c	Describe planned method of extracting data from reports (such as piloting forms, done independently, in duplicate), any processes for obtaining and confirming data from investigators	Data extraction and synthesis (pages 6-7)
Data items	12	List and define all variables for which data will be sought (such as PICO items, funding sources), any pre-planned data assumptions and	Data extraction and synthesis (pages 6-7)

Section and topic	Item No	Checklist item	Where they are addressed
		simplifications	
Outcomes and prioritization	13	List and define all outcomes for which data will be sought, including prioritization of main and additional outcomes, with rationale	NA
Risk of bias in individual studies	14	Describe anticipated methods for assessing risk of bias of individual studies, including whether this will be done at the outcome or study level, or both; state how this information will be used in data synthesis	NA
Data synthesis	15a	Describe criteria under which study data will be quantitatively synthesised	NA
	15b	If data are appropriate for quantitative synthesis, describe planned summary measures, methods of handling data and methods of combining data from studies, including any planned exploration of consistency (such as I^2, Kendall's τ)	NA
	15c	Describe any proposed additional analyses (such as sensitivity or subgroup analyses, meta-regression)	NA
	15d	If quantitative synthesis is not appropriate, describe the type of summary planned	Study will be synthesised using a narrative approach (page 7, para 3).
Meta-bias(es)	16	Specify any planned assessment of meta-bias(es) (such as publication bias across studies, selective reporting within studies)	NA
Confidence in cumulative evidence	17	Describe how the strength of the body of evidence will be assessed (such as GRADE)	NA

BMJ Open

Exploring the theory, barriers and enablers for patient and public involvement across health, social care and patient safety: A protocol for a systematic review of reviews.

Journal:	*BMJ Open*
Manuscript ID	bmjopen-2017-018426.R1
Article Type:	Protocol
Date Submitted by the Author:	06-Sep-2017
Complete List of Authors:	Ocloo, J; King's College London, Health Service & Population Research Department<br>Garfield, Sarah; Imperial College Healthcare NHS Trust, Centre for Medication Safety and Service Quality; University College London, School of Pharmacy<br>Dawson, Shoba; University College London Ear Institute, evidENT<br>Dean Franklin, Bryony; University College London School of Pharmacy; Imperial College Healthcare NHS Trust, Centre for Medication Safety and Service Quality
**Primary Subject Heading**:	Health services research
Secondary Subject Heading:	Evidence based practice
Keywords:	patient and public involvement, patient safety and quality, health and social care, theory, barriers and enablers

SCHOLARONE™ Manuscripts

Title Page

Exploring the theory, barriers and enablers for patient and public involvement across health, social care and patient safety: A protocol for a systematic review of reviews.

Dr Josephine Ocloo, Dr Sara Garfield, Ms Shoba Dawson, Prof Bryony Dean Franklin

Corresponding author:

Dr Josephine Ocloo, Centre for Implementation Science (CIS) part of the National Institute for Health Research (NIHR) Collaboration for Leadership in Applied Health Research and Care South London.
Health Service & Population Research Department
Institute of Psychiatry, Psychology & Neuroscience (IoPPN)
King's College London Room M306De Crespigny Park
Denmark Hill
London SE5 8AF
UK.
Email: Josephine.ocloo@kcl.ac.uk
Tel: +44 0 207 848 0115

Co—authors:

Dr Sara Garfield, Imperial College Healthcare NHS Trust, London, UK,
University College London School of Pharmacy, London, UK.

Ms Shoba Dawson
University College London, evidENT, Ear Institute,
London, UK.

Professor Bryony Dean Franklin, Imperial College Healthcare NHS Trust, London, UK
University College London School of Pharmacy
London, UK.

Key words:

Patient and public Involvement, theory barriers and enablers, patient safety and quality, health and social care.

Word count: 2,694 (excluding title page appendices and references)

For peer review only

Exploring the theory, barriers and enablers for patient and public involvement across health, social care and patient safety: A protocol for a systematic review of reviews.

Abstract

Introduction: The emergence of patient and public involvement (PPI) in healthcare in the UK can be traced as far back as the 1970's. More recently, campaigns by harmed patients and their relatives have emerged as a result of clinical failings in the NHS, challenging paternalistic healthcare, which have led to a new focus on PPI in quality and safety, nationally and internationally. Evidence suggests that PPI within patient safety is often atheoretical and located within a biomedical discourse. This review will explore the literature on PPI across patient safety, healthcare and social care to identify theory, barriers and enablers that can be used to develop PPI in patient safety.

Methods and analysis: Systematic searches of three electronic bibliographic databases will be conducted, using both MeSH and free-text terms to identify empirical literature published from database inception to May 2017. The screening process will involve input from at least two researchers and any disagreement will be resolved through discussion with a third reviewer. Initial inclusion and exclusion criteria have been developed and will be refined iteratively throughout the process. Data extraction from included articles will be conducted by at least two researchers using a data extraction form. Extracted information will be analysed using a narrative review approach, which synthesises data using a descriptive method.

Ethics and Dissemination: No ethical approval is required for this review as no empirical data was collected. We believe the findings and recommendations from this review will be particularly relevant for an audience of academics and policymakers. The findings will therefore be written up and disseminated in international peer reviewed journals and academic conferences with a health focus. They will also be disseminated to leading health policy organisations in the NHS, such as NHS England and NHS Improvement and national policy bodies such as the Health Foundation.

Keywords: patient and public Involvement, theory, barriers and enablers, patient safety and quality, health and social care.

For peer review only

For peer review only - http://bmjopen.bmj.com/site/about/guidelines.xhtml

Strengths and limitations of this study

Strengths

- This review will address a perceived knowledge and theory gap with PPI in patient safety.
- It will critically evaluate and synthesise evidence from across health, social care and patient safety.

Limitations

- The review will not include the grey literature.
- The review will focus solely on published peer-reviewed reviews.

Introduction:

The importance of involving patients, service users, carers and the public in the UK in health and social care and research has grown significantly in recent decades[1-4]. These developments have been linked to the growth in policy initiatives around citizenship, democracy and rights[5]. More recently the idea of partnership working with patients and the public has gained even more prominence as a result of serious clinical and service failings in the UK[6, 7], and internationally[8-10]. These initiatives have often been driven by the campaigns of patients who have experienced harm and their relatives, leading to a new focus on PPI and its importance in improving quality and safety within healthcare.

In the United Kingdom, a commitment to patient and public involvement (PPI) is now firmly enshrined in key legislation. This covers the Health and Social Care Act[11], the NHS Constitution[12] and the duty by NHS England (s13Q of the National Health Service Act 2006 (as amended by the Health and Social Care Act 2012) to properly involve patients and the public in its commissioning processes and decisions. In addition, key regulations set out essential standards of quality and safety that people who use health and adult social care services have a right to expect[13]. These rights are underpinned by wider but complimentary policy approaches such as public and patient experience and engagement (PPE). These approaches aim to place people who use services at the centre of care, to understand their experience of services, to empower them to make decisions and involve them in the design and delivery of care[14]. The NHS next stage review identified three strands of quality: patient experience, patient safety and clinical effectiveness. PPE approaches aim to ensure that patient experience sits as an equal partner in these three strands of quality[15].

These types of PPI initiatives are by no means unique to the UK and similar reforms in health and social care can be seen across a range of international settings[16-20].

Despite these policy developments, there have been an increasing number of criticisms about the nature of PPI in practice. There is uncertainty about how to do it well, in ways that constitute genuine partnerships and which involve a diversity of patients and the public, rather than a few selected individuals[21-23]. In particular, in the area of patient safety, the literature on PPI has been seen as dominated by a biomedical approach[24, 25], has been atheoretical[26] and has not addressed power inequities and discrimination[27]. This has exposed PPI to criticisms of exclusivity and tokenism[28].

For peer review only - http://bmjopen.bmj.com/site/about/guidelines.xhtml

This is seen as leading to a narrow model of PPI that fails to empower patients and the public in the involvement process. This gap is seen to be out of keeping with the wider literature on PPI more broadly in healthcare, which highlights the contested and bottom up nature and drivers for involvement and the way in which various global health social movements have provided collective challenges to poor care and discriminatory or paternalistic services and medical policy and belief systems[29]. These drivers have led to the development of theory, methods and approaches, particularly within mental health, that have been used to develop wider PPI models and methods based upon co-production and partnership. In patient safety, despite the campaigns by patients who have experienced harm and their relatives acting as a catalyst for the patient safety movement, there is evidence that suggests that lay members are struggling to influence decisions and are largely expected to work within existing systems in improving quality and safety[7,26]. Involvement at this level has therefore been criticised as providing little opportunity to influence decision-making processes in any depth, maintaining power differentials and the status quo.

This review is being undertaken to address a perceived knowledge and theory gap with PPI in patient safety. In order to address this the review will identify reviews of the published empirical literature, to look at: 'What are the theories, barriers and enablers in undertaking patient and public involvement in health social care and patient safety'?.

Terminology

There is considerable confusion about the use of terminology in this area. A number of different terms are often used synonymously with involvement, such as engagement or participation, whilst the terms patients and the public are also used interchangeably with 'citizen', 'consumer', 'layperson' or service user. These conceptual differences have emerged from disparate traditions social movements, policies and practices to describe the involvement process[30]. They have also been used to imply a greater or lesser level of involvement, power or influence in decision-making processes within an organisation. However, this language does not always reflect the underlying ethos of these involvement activities[31]. In the absence of a consensus on terminology, we define involvement as an activity that is done 'with' or 'by' patients or members of the public rather than 'to', 'about' or 'for' them[32]. This definition reflects the fact that the involvement process has increasingly come to be seen as a process of partnership: *"…the active participation of patients, carers, community representatives, community groups and the public in how services are planned, delivered and evaluated. It is broader and deeper than traditional consultation. It involves the ongoing process of developing and sustaining constructive relationships, building strong, active partnerships and holding a meaningful dialogue with stakeholders".* (p.9)[13]

Methods and analysis

Study design

This systematic review of reviews will be conducted and reported in accordance with the Preferred Reporting Items for Systematic Review and Meta-Analysis Protocols (PRISMA-P) (see Supplementary file S2)[33] and the results reported following the PRISMA guidelines[34]. The flow diagram (Figure 1) demonstrates the different stages of the systematic review and how these interact and influence one another.

For peer review only - http://bmjopen.bmj.com/site/about/guidelines.xhtml

Flow Diagram – Stages of the Systematic Review

Insert Figure 1

Study registration

Based on the PRISMA-P guidelines, this review was registered with the International Prospective Register of Systematic Reviews (PROSPERO) on 28/4/2017 (registration number CRD42017067848: (http://www.crd.york.ac.uk/PROSPERO/).

Eligibility criteria

Studies will be included in this review if they fulfil the following criteria:

Inclusion Criteria

1. **Type of study** – systematic reviews that focus on the concept of, or approaches to, PPI and/or PPE across patient safety, healthcare and social care.
2. **Setting** - any organisational setting (e.g. primary care, mental health, hospital, tertiary care, and voluntary etc)
3. **Type of involvement -** This review will focus largely on the collective level or what has also been referred to as public involvement [this literature generally relates to public involvement in strategic decisions in health services e.g. in service improvement planning, and/or organisational design and can cover various areas at a local or national level in governance, policy making, commissioning, monitoring, evaluation and research]. The review will however look at some examples of involvement in direct care, but only where this relates to activities to improve health, social care or patient safety and quality more widely. The literature covering public involvement is distinct from literature focussing on patient involvement which refers more specifically to 'the involvement of individual patients, together with health professionals, in making decisions (including shared decision-making) about their own care'[35]. The review will not include this much wider and more substantial body of literature on aspects or proxies for patient involvement, or engagement in their own clinical treatment such as shared decision- making and patient centeredness.
4. **Study design** - systematic reviews based on either published empirical studies (e.g. using quantitative, qualitative, or mixed methods), or theoretical papers. Where papers include both a systematic review and an empirical study, we will include data relating to the review if it is presented separately.

Articles that meet any of the following criteria will be excluded:

1. Systematic reviews that do not have a specific focus on PPI at the collective service improvement level.
2. Empirical studies
3. Non-systematic reviews
4. Reviews that focus on PPI at the individual level in treatment and decision-making.
5. Reviews not written in English

Search strategy

Three main electronic bibliographic databases including MEDLINE, EMBASE and PsycINFO will be searched for potential studies from the inception of databases to May 2017 as described in Table 1.

For peer review only - http://bmjopen.bmj.com/site/about/guidelines.xhtml

Table 1: A structured search of key electronic bibliographic databases:

Search type:	Database:	Database history:	Filters applied:
Systematic review of the literature	(MEDLINE (via OVID 1946 to May 2017)	Biomedical database produced by the National Library of Medicine, with over 3000 journals	Limited to systematic reviews, narrative reviews, bibliometric reviews and meta-analysis
	EMBASE (1974 to May 2017	Biomedical and pharmacological database, with a more European focus	Limited to systematic reviews, narrative reviews, bibliometric reviews and meta
	PsycINFO (1967 to May 2017).	Produced by the American Psychological Association covering over 1300 journals from Psychology and related disciplines	Limited to systematic reviews, narrative reviews, bibliometric reviews and meta

A comprehensive search strategy will include a combination of five main blocks of terms including and relating to public involvement **(**public, patient, carer, consumer, citizen, lay, service user, stakeholder, family, relative, survivor); type of involvement (involvement, collaboration, engagement, partnership, consultation, participation, user-led, consumer or patient panel, advisory board/group/panel); health and social care setting (health services, health care, social care, public health, mental health etc); patient safety (safe*, adverse event$, error* etc) and type of review (systematic, narrative, meta, bibliometric); using a combination of MeSH (Medical Subject Headings) terms and free text (see Supplementary file S1: example search strategy). Terms used to describe public involvement and type of involvement were similar to other studies[36,37]. Database searches will be supplemented by citation searches and reference lists of included studies. Additionally, several scoping exercises in these electronic databases will be applied to maximise the sensitivity and specificity of the developed search strategy.

Screening of studies

An Endnote library will be utilised to combine and export the results of the searches from different databases. Duplicates will be removed prior to the selection of studies. Study selection will be completed in two stages. Firstly, titles and abstracts will be screened in order to identify eligible and relevant studies, followed by full-text screening to identify reviews eligible for inclusion.

Abstract and title screening

Abstracts and titles will be used to initially screen and identify eligible and relevant studies using the selection criteria above for the articles remaining in the literature review. A random sample of 10% titles and abstracts will be independently screened by both the lead researcher (JO) and a secondary reviewer (SG or SD). Any disagreements will be resolved through discussions until consensus is reached. However, if we are unable to reach consensus, disagreements will be resolved through discussions with an independent third reviewer (BDF). Once a consensus is reached for the 10% sample, the remaining titles and abstracts will be screened by the lead researcher alone.

For peer review only - http://bmjopen.bmj.com/site/about/guidelines.xhtml

Full-text screening

At the outset of this process a 10% sample of full-text papers will be screened and reviewed independently by both the lead researcher and a second reviewer (SG or SD). Any disagreements will be resolved through discussion or involving a third reviewer. If necessary a further 10% of papers will be independently reviewed to test for agreement and then the remaining reviews will be screened by the lead researcher (JO) alone. Along with the lead researcher, SG and SD will screen all included papers to ensure they meet the inclusion criteria.

Data extraction and synthesis:

A data extraction form will be used (see Supplementary file S1), to focus on the characteristics that are relevant to this study. The form will be piloted on 10% of randomly selected studies. Any necessary modifications will then be made to the data extraction tool. Data from the included studies will be extracted and categorised using the following headings below.

- Study characteristics- authors, year, study aims, type of study/study design, health topic focus and setting
- Participant characteristics- types of people involved (including age, ethnicity, gender etc)
- Evidence of equality and diversity (in accordance with the Equality and Human Rights Act and NHS Constitution)
- Definition(s) of PPI
- Methods of PPI (involvement can be seen as a spectrum, with a range of service user involvement activities that can take place at multiple levels, i.e. consultation, collaboration or user control)[38, 39]
- Use of theory (to understand, analyse, describe, and/or facilitate PPI activities)
- Barriers and facilitators to involvement.
- Reported impacts of PPI

The data extraction process will be carried out by both the lead researcher and a secondary reviewer (SG or SD) as per the earlier processes. Any disagreements will be resolved through discussion or involving a third reviewer.

A systematic narrative approach will be used for synthesising the results of this review, due to the heterogeneous nature of the included published empirical literature[40]. This will include familiarisation with the papers and identifying data relating to the themes linked to study aims and outcomes. Data will be summarised in a descriptive form in order to allow us to draw conclusions about the available evidence.

For peer review only - http://bmjopen.bmj.com/site/about/guidelines.xhtml

Quality assessment

A quality assessment is normally conducted in systematic reviews using quality assessment checklists such as Critical Appraisal Skills Programme[1] (CASP) to assess the quality of individual studies. As this is a systematic review of reviews, an empirically developed instrument for documenting the methodological quality of systematic reviews: Assessment of Methodological Quality of Systematic Reviews (AMSTAR)[2], will be used in this study to determine if eligible reviews meet the minimum criteria based on quality.

Ethics and dissemination

No ethical approval is required for this review as it is a systematic review and does not involve the collection of primary data.

We believe the findings and recommendations from this review will be particularly relevant for an audience of academics and policymakers given its focus across different sectors of healthcare. The findings will therefore be written up and published in international peer reviewed journals as well as disseminated through academic conferences with a health focus in-line with suggestions made by the Guidance for Reporting Involvement of Patients and the Public (GRIPP2)[41].

The review however, will also be used to provide opportunities for testing methods of PPI in practice in patient safety and evaluating its impact in improving services. The findings will therefore be disseminated via a range of policy organisations such as NHS England and NHSE Improvement as well as national policy bodies such as the Health Foundation and its Q Network. Q is an initiative connecting people, who have improvement expertise, across the UK. It is led by the Health Foundation and supported and co-funded by NHS Improvement.

---

[1] http://www.casp-uk.net/casp-tools-checklists

[2] https://amstar.ca/Amstar_Checklist.php

For peer review only - http://bmjopen.bmj.com/site/about/guidelines.xhtml

Contributorship: JO (first author) developed the intellectual idea for the review and led on all of the work in drafting the protocol and its various components. SG and SD (second and third authors) provided detailed input concerning the study design and methods and analysis for the protocol and SD in addition to this and alongside JO developed the search strategy. BDF contributed to the intellectual development of the protocol, commenting on ideas, drafts and in making suggestions and helping to resolve disagreement and reach consensus. JO, SG, SD and BDF were all involved in the drafting of the protocol and approval for publication.

Competing Interests

None

Funding

The Health Foundation, National Institute for Health Research (NIHR)

Disclaimer

The research is supported by the Centre for Implementation Science (CIS) which is part of the National Institute for Health Research (NIHR) Collaboration for Leadership in Applied Health Research and Care South London at King's College Hospital NHS Foundation Trust and the National Institute for Health Research (NIHR) Imperial Patient Safety Translational Research Centre. The views expressed are those of the author(s) and not necessarily those of the NHS, the NIHR or the Department of Health.

For peer review only - http://bmjopen.bmj.com/site/about/guidelines.xhtml

References

1. Department of Health. Patient and Public Involvement in the NHS. London: Department of Health. 1999.

2. Department of Health. Real Involvement: Working with People to Improve Services. London: Department of Health. 2008.

3. Department of Health. Equity and Excellence: Liberating the NHS. London: Department of Health. 2010.

4. Department of Health. Framework for patient and public participation in public health commissioning. London: Department of Health. 2017.

5. Newman J. Modernising Governance: New Labour, Policy and Society. London: Sage; 2001.

6. Bristol Royal Infirmary Inquiry. The Report of the Public Inquiry into Children's Heart surgery at the Bristol Royal Infirmary 1984–1995. London: HMSO, 2001.

7. Francis R. Report of the Mid Staffordshire NHS Foundation Trust Public Inquiry. London: Stationery Office, 2013.

8. Kohn LT, Corrigan JM, Donaldson MS. To err is human: building a safer health system. Washington: National Academies Press, 1999.

9. Walshe K, Shortell SM. When things go wrong: how Health Care Organizations deal with major failures. Health Aff (Millwood) 2004; 23: 103–11.

10. WHO. World Alliance for Patient Safety forward programme. Geneva: World Health Organization, 2008–2009.

11. Department of Health. Research Governance Framework for Health and Social Care. 2005. https://www.gov.uk/government/publications/research-governance-framework-for-health-and-social-care-second-edition. Accessed 12 Jan 2017.

12. Department of Health. NHS Constitution for England. London: Department of Health. 2012. https://www.gov.uk/government/publications/the-nhs-constitution-for-england

For peer review only - http://bmjopen.bmj.com/site/about/guidelines.xhtml

13. Care Quality Commission: Guidance about compliance Essential standards of quality and safety March 2010.

14. Department of Health. Putting people at the heart of care. London: Department of Health. 2009. http://webarchive.nationalarchives.gov.uk/20130107105354/http:/www.dh.gov.uk/en/Publicationsandstatistics/Publications/PublicationsPolicyAndGuidance/DH_106038

15. Darzi A. High Quality Care For All: NHS Next Stage Review Final Report. London: Department of Health. 2009.

16. Charles C and DeMaio S. Lay participation in health care decision-making: a conceptual framework. Journal of Health Politics, Policy and Law, 1993

17. Church J, Saunders D, Wanke M et al. Citizen participation in health decision-making: past experience and future prospects. Health Policy 2002; 23:12–32.

18. Tritter J.Q. Revolution or evolution: the challenges of conceptualizing patient and public involvement in a consumerist world, Health Expectations, 2009; 12:3, 275–87.

19. Coulter, A. Engaging Patients in Healthcare. New York: McGraw Hill Professional. 2013.

20. Carman KL, Dardess P, Maurer M et al. Patient and family engagement: a framework for understanding the elements and developing interventions and policies. Health Affairs (Millwood) 2013; 32:223–31.

21. National Institute for Health Research. Going the Extra Mile: improving the Nation's health and wellbeing through public involvement in research. London: National Institute for Health Research, 2015.

22. Beresford P. Beyond the usual suspects. London: Shaping Our Lives. 2013.

23. Martin GP. Representativeness, legitimacy and power in public involvement in health-service management. Social Science and Medicine 2008; 67:1757–65.

24. Sharpe VA and Faden AI. Medical Harm. Cambridge: Cambridge University Press. 2001.

25. Ocloo JE. Harmed patients gaining voice: challenging dominant perspectives in the construction of medical harm and patient safety reforms. Social Science and Medicine 2010; 71:510–16.

26. Peat M, Entwistle V, Hall J, Birks Y, Golder G. Scoping review and approach to appraisal of interventions intended to involve patients in patient safety. Journal of Health Services Research & Policy, 2010; 15: 17–25.

27. Ocloo JE, Fulop NJ. Developing a critical approach to patient and public involvement in patient safety in the NHS: learning lessons from other parts of the public sector? Health Expect 2012; 15:424–32.

28. Ocloo J, Matthews R. BMJ Quality and Safety 2016; 0:1–7. doi:10.1136/bmjqs-2015-004839

For peer review only - http://bmjopen.bmj.com/site/about/guidelines.xhtml

29. Brown P, Zavestoski S. Social movements in health: an introduction. Sociology of Health and Illness 2004; 26: 679–94.

30. Gibson A, Britten N, Lynch J. Theoretical directions for an emancipatory concept of patient and public involvement. Health (London) 2012; 16: 531–47.

31. Faulkner A, Yiannoullou S, Kalathil J, et al. Involvement for Influence. 4PI National Involvement Standards. London: National survivor User Network (NSUN), 2015.

32. INVOLVE. Briefing notes for researchers: involving the public in NHS, public health and social care research. Eastleigh: Hampshire INVOLVE, 2012.

33. Shamseer L, Moher D, Clarke M. et al. PRISMA-P Group. Preferred reporting items for systematic review and meta-analyses protocols (PRISMA-P) 2015: elaboration and explanation. BMJ. 2015; 349 :g7647. doi:10.1136/bmj.g7647

34. Moher D, Liberati A, Tetzlaff J, Altman DG, The PG. Preferred Reporting Items for Systematic Reviews and Meta-Analyses: The PRISMA Statement. PLoS Medicine. 2009; 6(7):e1000097.

35. Florin D, Dixon J. Public involvement in health care. British Medical Journal, 2004; 328: 159–161.

36. Brett J, Staniszewska S, Mockford C, Seers K, Herron-Marx S, Bayliss H. The PIRICOM Study: A Systematic Review of the Conceptualisation, Measurement, Impact and Outcomes of Patients and Public Involvement in Health and Social Care Research. London: United Kingdom Clinical Research Council, 2010.

37. Rogers M, Bethel A, Boddy K. Development and testing of a medline search filter for identifying patient and public involvement in health research. Health Information and Libraries Journal, 2017; 34:125-133.

38. Carman KL, Dardess P, Maurer M, et al. Patient and family engagement: a framework for understanding the elements and developing interventions and policies. Health Affairs (Millwood), 2013; 32: 223–231.

39. Gibson, A., Welsman, J. and Britten, N. Evaluating patient and public involvement in health research: from theoretical model to practical workshop. Health Expectations. doi:10.1111/hex.12486.

40. Popay J, Roberts H, Sowden A, Petticrew M, Arai L, Rodgers M, Britten N, Roen K and Duffy S. Guidance on the Conduct of Narrative Synthesis in Systematic Reviews: A Product from the ESRC Methods Programme, 2006.

41. Staniszewska S, Brett J, Simera I et al. GRIPP2 reporting checklists: tools to improve reporting of patient and public involvement in research. BMJ, 2017; 358: j3453.

For peer review only - http://bmjopen.bmj.com/site/about/guidelines.xhtml

For peer review only

For peer review only - http://bmjopen.bmj.com/site/about/guidelines.xhtml

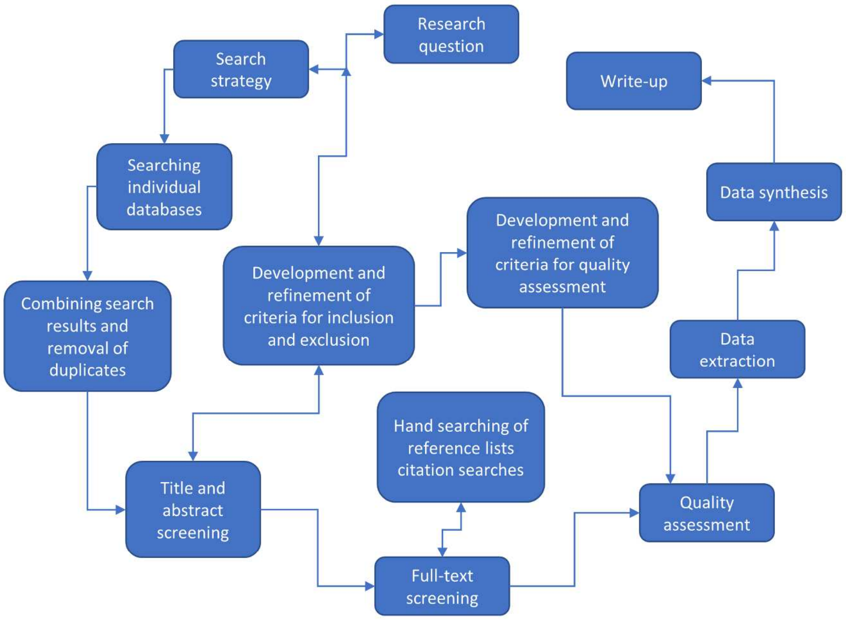

209x140mm (300 x 300 DPI)

view only

Appendix 1: Example search strategy Medline

Searches	Results
consumer participation/	15860
patient participation/	22136
or/1-2	37690
(patient* adj1 (participat* or involv* or engag* or partnership or partners or collaborat* or consult*)).ab,ti.	24412
(public adj1 (participat* or involv* or engag* or partnership or partners or collaborat* or consult*)).ab,ti.	1982
(user* adj1 (participat* or involv* or engag* or partnership or partners or collaborat* or consult*)).ab,ti.	1416
(service user* adj1 (participat* or involv* or engag* or partnership or partners or collaborat* or consult*)).ab,ti.	312
(consumer* adj1 (participat* or involv* or engag* or partnership or partners or collaborat* or consult*)).ab,ti.	895
(lay adj1 (participat* or involv* or engag* or partnership or partners or collaborat* or consult*)).ab,ti.	141
(citizen* adj1 (participat* or involv* or engag* or partnership or partners or collaborat* or consult*)).ab,ti.	404
(carer* adj1 (participat* or involv* or engag* or partnership or partners or collaborat* or consult*)).ab,ti.	281
(caregiver* adj1 (participat* or involv* or engag* or partnership or partners or collaborat* or consult*)).ab,ti.	844
(customer* adj1 (participat* or involv* or engag* or partnership or partners or collaborat* or consult*)).ab,ti.	45
(client* adj1 (participat* or involv* or engag* or partnership or partners or collaborat* or consult*)).ab,ti.	746
(community* adj1 (participat* or involv* or engag* or partnership or partners or collaborat* or consult*)).ab,ti.	7297
(stakeholder* adj1 (participat* or involv* or engag* or partnership or partners or collaborat* or consult*)).ab,ti.	2069
((patient* and public) adj1 (involv* or participat* or engag* or partnership or partners or collaborat* or consult*)).ab,ti.	6090

For peer review only - http://bmjopen.bmj.com/site/about/guidelines.xhtml

(user led or user-led or lay control or user control).ab,ti.	201
((representative* or patient representative* or patient advocate* or expert by experience or famil* or relative* or survivor*) adj1 (participat* or involv* or engag* or partnership or partners or collaborat* or consult*)).ab,ti.	6905
((patient* or consumer* or citizen* or advisory) adj1 board*).ab,ti.	1439
((patient* or consumer* or citizen* or advisory) adj1 group*).ab,ti.	75334
((patient* or consumer* or citizen* or advisory) adj1 panel*).ab,ti.	1215
(citizen* adj1 (jury or juries)).ab,ti.	64
or/4-23	128820
3 or 24	159935
health services research.mp. or exp health services research/	151453
social care research.mp.	28
social service*.mp.	8561
public health.mp. or exp public health/	6876537
psychology.mp. or exp psychology/	276350
psychiatry.mp. or exp psychiatry/	122746
sociology.mp. or exp sociology/	1228269
nursing.mp. or exp nursing/	505413
(mental health or mental healthcare).mp. or exp mental health/	135802
secondary care.mp. or exp secondary care/	4285
tertiary care.mp. or exp tertiary healthcare/	33981
or/26-36	8022042
Family Practice/	64218
Primary Health Care/	65710
Physicians, Family/	15936
Community Health Services/	29975
Community Dentistry/	1198
Community Health Nursing/	19280
Community Mental Health Services/	18322
Community Pharmacy Services/	3625
Home Care Services/	31334

Community Mental Health Centers/	2918
family pract$.tw.	8627
general practice$.tw.	34098
community based.tw.	41862
community care.tw.	3486
family medicine.tw.	7419
family physician$.tw.	11393
primary care.tw.	81635
(primary health care or primary healthcare).tw.	18862
family doctor$.tw.	3837
primary medical care.tw.	805
general physician$.tw.	1361
general practitioner$.tw.	40695
primary care practitioner$.tw.	1148
(community adj (health or healthcare or health care)).tw.	16890
primary healthcare team$.tw.	101
primary health care team$.tw.	492
primary medical care team$.tw.	1
practice nurse$.tw.	4672
practice manager$.tw.	374
(gpsi or gpwsi).tw.	30
(practitioner$ adj3 special interest$).tw.	68
(primary care or primary health care or general practice or family practice or family medicine).nw.	40736
or/38-69	369470
health care.mp. or "Delivery of Health Care"/	651145
(health care services or health care delivery).mp.	18161
37 or 71 or 72	8180235
70 or 73	8253391
patient safety.mp. or Patient Safety/	26848
Safety Management/	18778

Medication Errors/ or Patient Harm/ or Medical Errors/	26909
patient risk.mp.	2561
Safety/ or safety.mp.	388412
Adverse event$.mp.	102273
adverse drug event$.mp.	2514
incident$.mp.	96270
error$.mp.	271931
error*.mp.	271931
incident*.mp.	96270
near miss*.mp.	1578
fall*.mp.	196678
slip*.mp.	14834
trip*.mp.	306863
75 or 76 or 77 or 78 or 79 or 80 or 81 or 82 or 83 or 84 or 85 or 86 or 87 or 88 or 89	1288414
74 or 90	8892947
25 and 91	125154
((meta or narrative or bibliometric or systematic) and review).ab,ti.	106442
exp "review"/ or review.ab,ti.	2552853
93 or 94	2552853
92 and 93	2052

only

Appendix 2: Data Extraction Form

Data Extraction Form	Details
Citation ID (author, year),	
Systematic review aims	
Setting (primary, secondary, social and community care etc)	
Countries where the research conducted	
Health topic focus	
Sample Size and Participant characteristics (younger people, over 65, ethnicity, general public etc) [This should include the final number of studies reported on and participant characteristics (e.g. pre-defined inclusion criteria or characteristics reported on) including sample size if reported]	
Evidence of Equality and Diversity<br><br>*(Equality defined in line with the protected characteristics covered by the Equality &Human Rights Act & NHS Constitution e.g age; disability; gender reassignment; marriage & civil partnership; pregnancy & maternity; race; religion or belief; sex; sexual orientation),*<br><br>**Diversity** literally means difference. When it is used as a contrast or addition to equality, it is about recognising individual as well as group differences, treating people as individuals, and placing positive value on diversity in the community and in the workforce.	
Definition(s) of PPI	
Methods of PPI involvement<br>Involvement can be seen as a spectrum, with a range of service user involvement activities that can take place at multiple levels.<br><br>**Information:** Service users are told what is happening, they have no influence over the decision-making.<br>**Consultation:** Service users are asked their views. They have limited influence decision-making.<br>**Participation:** Service users' views are sought and taken into account. They have a direct impact on decision-making.<br>**Partnership/ co-production**: Working as equals, service users share decisions and responsibility, influencing and determining outcomes.<br>**Control:** Service users control the decision-making.	
Theories, frameworks and concepts and how they are used/defined (e.g. to understand, analyse, describe, facilitate, approach PPI activities)	
Evidence of barriers and facilitators to involvement	
Evidence of impacts of PPI	

For peer review only

PRISMA-P (preferred reporting items for systematic review and meta-analysis protocols) checklist: recommended items to address in a systematic review protocol[33]

Section and topic	Item No	Checklist item	Where they are addressed
**Administrative information**			
Title:			
Identification	1a	Identify the report as a protocol of a systematic review	Title page
Update	1b	If the protocol is for an update of a previous systematic review, identify as such	NA
Registration	2	If registered, provide the name of the registry (such as PROSPERO) and registration number	PROSPERO CRD42017067848 (pg.4, study registration)
Authors:			
Contact	3a	Provide name, institutional affiliation, e-mail address of all protocol authors; provide physical mailing address of corresponding author	Title page
Contributions	3b	Describe contributions of protocol authors and identify the guarantor of the review	Page 13
Amendments	4	If the protocol represents an amendment of a previously completed or published protocol, identify as such and list changes; otherwise, state plan for documenting important protocol amendments	NA
Support:			
Sources	5a	Indicate sources of financial or other support for the review	Page 13
Sponsor	5b	Provide name for the review funder and/or sponsor	Health Foundation, NIHR, page 13
Role of sponsor or funder	5c	Describe roles of funder(s), sponsor(s), and/or institution(s), if any, in developing the protocol	NA
**Introduction**			
Rationale	6	Describe the rationale for the review in the context of what is already known	Page 3, second paragraph

Section and topic	Item No	Checklist item	Where they are addressed
Objectives	7	Provide an explicit statement of the question(s) the review will address with reference to participants, interventions, comparators, and outcomes (PICO)	Aim of the review and research question (page 3, second para). This does not include reference to specific participants, interventions comparators and outcomes as the review does not focus on specific interventions.
**Methods**			
Eligibility criteria	8	Specify the study characteristics (such as PICO, study design, setting, time frame) and report characteristics (such as years considered, language, publication status) to be used as criteria for eligibility for the review	Inclusion and Exclusion criteria (page 4)
Information sources	9	Describe all intended information sources (such as electronic databases, contact with study authors, trial registers or other grey literature sources) with planned dates of coverage	Search strategy pages page 5, Table 1
Search strategy	10	Present draft of search strategy to be used for at least one electronic database, including planned limits, such that it could be repeated	Appendix 1: (pages 8-11)
Study records:			
Data management	11a	Describe the mechanism(s) that will be used to manage records and data throughout the review	Screening of studies (page 5 and 6)
Selection process	11b	State the process that will be used for selecting studies (such as two independent reviewers) through each phase of the review (that is, screening, eligibility and inclusion in meta-analysis)	Abstract and Title screening and Full-text screening (page 5)
Data collection process	11c	Describe planned method of extracting data from reports (such as piloting forms, done independently, in duplicate), any processes for obtaining and confirming data from investigators	Data extraction and synthesis (pages 6)

Section and topic	Item No	Checklist item	Where they are addressed
Data items	12	List and define all variables for which data will be sought (such as PICO items, funding sources), any pre-planned data assumptions and simplifications	Data extraction and synthesis (pages 6)
Outcomes and prioritization	13	List and define all outcomes for which data will be sought, including prioritization of main and additional outcomes, with rationale	NA
Risk of bias in individual studies	14	Describe anticipated methods for assessing risk of bias of individual studies, including whether this will be done at the outcome or study level, or both; state how this information will be used in data synthesis	NA
Data synthesis	15a	Describe criteria under which study data will be quantitatively synthesised	NA
	15b	If data are appropriate for quantitative synthesis, describe planned summary measures, methods of handling data and methods of combining data from studies, including any planned exploration of consistency (such as I^2, Kendall's τ)	NA
	15c	Describe any proposed additional analyses (such as sensitivity or subgroup analyses, meta-regression)	NA
	15d	If quantitative synthesis is not appropriate, describe the type of summary planned	Study will be synthesised using a narrative approach (page 6, para 4).
Meta-bias(es)	16	Specify any planned assessment of meta-bias(es) (such as publication bias across studies, selective reporting within studies)	NA
Confidence in cumulative evidence	17	Describe how the strength of the body of evidence will be assessed (such as GRADE)	NA

PRISMA-P (preferred reporting items for systematic review and meta-analysis protocols) checklist: recommended items to address in a systematic review protocol[32]

Section and topic	Item No	Checklist item	Where they are addressed
**Administrative information**			
Title:			
Identification	1a	Identify the report as a protocol of a systematic review	Title page (Page 1)
Update	1b	If the protocol is for an update of a previous systematic review, identify as such	NA
Registration	2	If registered, provide the name of the registry (such as PROSPERO) and registration number	PROSPERO CRD42017067848 (pg.4, study registration)
Authors:			
Contact	3a	Provide name, institutional affiliation, e-mail address of all protocol authors; provide physical mailing address of corresponding author	Title page (Page 1)
Contributions	3b	Describe contributions of protocol authors and identify the guarantor of the review	Page 14
Amendments	4	If the protocol represents an amendment of a previously completed or published protocol, identify as such and list changes; otherwise, state plan for documenting important protocol amendments	NA
Support:			
Sources	5a	Indicate sources of financial or other support for the review	Page 14
Sponsor	5b	Provide name for the review funder and/or sponsor	Health Foundation, NIHR, page 14
Role of sponsor or funder	5c	Describe roles of funder(s), sponsor(s), and/or institution(s), if any, in developing the protocol	NA

Section and topic	Item No	Checklist item	Where they are addressed
**Introduction**			
Rationale	6	Describe the rationale for the review in the context of what is already known	Page 4, third paragraph
Objectives	7	Provide an explicit statement of the question(s) the review will address with reference to participants, interventions, comparators, and outcomes (PICO)	Aim of the review (page 4)
**Methods**			
Eligibility criteria	8	Specify the study characteristics (such as PICO, study design, setting, time frame) and report characteristics (such as years considered, language, publication status) to be used as criteria for eligibility for the review	Inclusion and Exclusion criteria (page 5)
Information sources	9	Describe all intended information sources (such as electronic databases, contact with study authors, trial registers or other grey literature sources) with planned dates of coverage	Search strategy pages 5-6 (Table 1)
Search strategy	10	Present draft of search strategy to be used for at least one electronic database, including planned limits, such that it could be repeated	Appendix 1: (pages8-12)
Study records:			
Data management	11a	Describe the mechanism(s) that will be used to manage records and data throughout the review	Screening of studies (page 6)
Selection process	11b	State the process that will be used for selecting studies (such as two independent reviewers) through each phase of the review (that is, screening, eligibility and inclusion in meta-analysis)	Abstract and Title screening and Full-text screening (page 6)
Data collection process	11c	Describe planned method of extracting data from reports (such as piloting forms, done independently, in duplicate), any processes for obtaining and confirming data from investigators	Data extraction and synthesis (pages 6-7)
Data items	12	List and define all variables for which data will be sought (such as PICO items, funding sources), any pre-planned data assumptions and	Data extraction and synthesis (pages 6-7)

Section and topic	Item No	Checklist item	Where they are addressed
		simplifications	
Outcomes and prioritization	13	List and define all outcomes for which data will be sought, including prioritization of main and additional outcomes, with rationale	NA
Risk of bias in individual studies	14	Describe anticipated methods for assessing risk of bias of individual studies, including whether this will be done at the outcome or study level, or both; state how this information will be used in data synthesis	NA
Data synthesis	15a	Describe criteria under which study data will be quantitatively synthesised	NA
	15b	If data are appropriate for quantitative synthesis, describe planned summary measures, methods of handling data and methods of combining data from studies, including any planned exploration of consistency (such as I^2, Kendall's τ)	NA
	15c	Describe any proposed additional analyses (such as sensitivity or subgroup analyses, meta-regression)	NA
	15d	If quantitative synthesis is not appropriate, describe the type of summary planned	Study will be synthesised using a narrative approach (page 7, para 3).
Meta-bias(es)	16	Specify any planned assessment of meta-bias(es) (such as publication bias across studies, selective reporting within studies)	NA
Confidence in cumulative evidence	17	Describe how the strength of the body of evidence will be assessed (such as GRADE)	NA
